# Supplementary material for: Reasons for and Behavioral Consequences of Male Dog Castration—A Questionnaire Study in Poland
Source: Animals (Basel). 2022 Jul 23;12(15):1883. doi: 10.3390/ani12151883 (PMC9330254; doi:10.3390/ani12151883)
Supplement: Supplementary file 1 [file animals-12-01883-s001.zip › animals-1799177-supplementary.pdf]

Table S1. Survey questions

Signalment questions

1. What is your gender?
2. What breed of dog/dogs do you own, possibly non-purebred?
3. What is the dog's age?
4. At what age was your dog castrated?
5. Reasons for castration:

Select all answers that apply:

a) roaming
b) aggression
c) health problems
d) disease prevention
e) birth control
f) hyperactivity
g) mounting
h) marking
i) personal conviction regarding castration
j) other

Questions related to aggression

6. Was your dog aggressive towards people before castration?

Possible answers:

a) yes
b) no
c) sometimes

7. Was your dog aggressive towards other dogs before castration?

Possible answers:

a) yes
b) no
c) sometimes

8. Was your dog aggressive towards other animals (other pets, etc.) before castration?

Possible answers:

a) yes
b) no
c) sometimes

9. Is the dog still aggressive towards people?

Possible answers:

a) yes
b) no
c) sometimes

10. Is the dog still aggressive towards other dogs?

Possible answers:

a) yes
b) no
c) sometimes

11. Is the dog still aggressive towards other animals (other pets, etc.)?

Possible answers:

a) yes
b) no
c) sometimes

Questions related to anxious behaviors

12. Did your dog present with anxious behaviors (e.g. hiding, freezing, running away, fear aggression, etc.) before castration?

Possible answers:

a) yes
b) no

13. If so, what were they?
14. Does your dog still present with anxious behaviors (e.g. hiding, freezing, running away, fear aggression, etc.)?

Possible answers:

a) yes

b) no
15. If so, what are they?
Questions related to roaming
16. Did your dog have a tendency to roam before castration?
Possible answers:
a) yes
b) no
17. Does your dog still have a tendency to roam?
Possible answers:
a) yes
b) no
Questions related to mounting
18. Did your dog have a tendency to mount before castration?
Possible answers:
a) yes
b) no
19. Does your dog still have a tendency to mount?
Possible answers:
a) yes
b) no
Questions related to over-marking
20. Did your dog have a tendency to over-mark objects before castration?
Possible answers:
a) yes
b) no
21. Does your dog still have a tendency to over-mark objects?
Possible answers:
a) yes
b) no
Questions related to dog's activity
22. Before castration, how would you rate your dog's activity levels?
Possible answers:
a) lethargic
b) somewhat active
c) moderately active
d) active
e) hyperactive
23. How would you rate your dog's activity levels at present?
Possible answers:
a) lethargic
b) somewhat active
c) moderately active
d) active
e) hyperactive
